# Thermo-Mechanical Behavior of Textile Heating Fabric Based on Silver Coated Polymeric Yarn

**DOI:** 10.3390/ma6031072

**Published:** 2013-03-20

**Authors:** Syed Talha Ali Hamdani, Prasad Potluri, Anura Fernando

**Affiliations:** Textiles Research Group, School of Materials, The University of Manchester, Manchester M13 9PL, UK; E-Mails: talha.hamdani@postgrad.manchester.ac.uk (S.T.A.H.); prasad.potluri@manchester.ac.uk (P.P.)

**Keywords:** elector-textile, silver, polymeric yarn, thermo-mechanical behavior, wearable, heating, knitted, antifreeze, protective clothing

## Abstract

This paper presents a study conducted on the thermo-mechanical properties of knitted structures, the methods of manufacture, effect of contact pressure at the structural binding points, on the degree of heating. The test results also present the level of heating produced as a function of the separation between the supply terminals. The study further investigates the rate of heating and cooling of the knitted structures. The work also presents the decay of heating properties of the yarn due to overheating. Thermal images were taken to study the heat distribution over the surface of the knitted fabric. A tensile tester having constant rate of extension was used to stretch the fabric. The behavior of temperature profile of stretched fabric was observed. A comparison of heat generation by plain, rib and interlock structures was studied. It was observed from the series of experiments that there is a minimum threshold force of contact at binding points of a knitted structure is required to pass the electricity. Once this force is achieved, stretching the fabric does not affect the amount of heat produced.

## 1. Introduction

Smart textiles refer to the emergence of electronic components with advanced fibers, polymers, yarns and fabrics. They sense the information about its wearer’s body and inform the wearer of the conditions of the body or send the information to the outside world [1]. The emergence of devices and miniaturized electronic apparatus has a great influence on modern people’s life patterns. As an increased number of people carry many portable devices, their clothing was also influenced by the changing lifestyle. The early smart clothing provided space in the clothes needed to carry portable devices conveniently as well as wire-guides (or path) for earphones.

To carry out these functions, smart or intelligent textiles must possess special properties that the conventional fiber does not have. The clothing must have a sensing function in order for it to perceive such variables as biomedical signals and body temperature of its wearer. Furthermore it must also have the actuator function to inform its wearer of the information or services available in the external world. It would be an ideal case if the fiber itself becomes the sensor or has a built-in actuator function.

For the electronic objects to co-exist with textile in the clothing environment despite the numerous difficulties and to implement common functions, they need an electro textile platform, which is defined as the infrastructure to be shared with electronics and textiles. Integration of the electronic products into fabric products is very difficult because of their manufacturing processes [2,3] and their physical properties that differ greatly: fabric is flexible and most electronics components are solids. Although smart textiles are progressing very rapidly [4,5] the connectivity, materials, fabrication and wear are still challenges to smart textiles [6]. To meet the requirements of design and functions with heterogeneous materials is a very complicated challenge where clothing designers, pattern designers, merchandisers, sewers, electronic engineers, and marketing specialists would have to agonize over a number of difficult problems [7].

Smart textiles that are manufactured mainly for the purpose of protection are referred to as protective textiles such as to provide thermal comfort and antifreeze safety. Wool fibers stuffed into crude footwear was the first nonwoven felts used for the protection of human feet [8]. Wool is the best natural occurring heat generating fiber that has been used to warm up the body in colder environments since ancient ages. Heat is released from the wool as it absorbs moisture. If 1 kg of dry wool is allowed to get saturated in humid air, 960 kJ [9] of heat will be generated that is equal to the heat produced by an electric blanket running for eight hours [10].

Metallic wires are also used in heated fabrics and personal heating garments. Electrical wires have been used in electrically heated wearing apparel and in heating gloves. The gloves can be worn with outer cape-leather for protection to skin from electrical wires [11,12]. The first documented evidence for the use of metallic wires in textile clothing is found in World War II [13]. Now-a-days, more sophisticated conductive yarns are being produced instead of metallic wires that contain the properties of textile yarns [14]. Manufacturing of conductive yarns helped textiles find application in the field of electrical components and electronics. Further textile actuators like heating fabrics have been used in numerous and varied fields such as sports, leisure, medical and automotive [15,16]. Smart clothing is being made with conductive yarns where an electrical current is required to pass through the fabric [17]. Heating Textiles can also be used for household use, such as to heated floors, walls and roof,* etc.* These textiles are also used for industrial or technical purposes such as motorbike gloves, heating pads, leisure garments and sport garments. Heating textiles are also used in the automobiles industry. It is also used in medical fields such as electrotherapy treatment, medical blanket for maintaining patient’s body temperature [18], strain sensors and motion capturing devices. Many accidents reported in the past years are due to accumulation of ice on aircrafts [19,20,21,22]. Heated textiles can also be used in the aircraft industry as an anti-freezing agent to avoid the accumulation of ice on the wings of aircraft.

Temperature control is one of the most important functions of clothes. Most of the heating elements use the principle of Joule’s heat, which is generated when an electric current is passed through a conductive material. All conductive materials are heating elements in principle. Conductive materials such as metals and conducting polymers are already being used in many textile applications such as antistatic materials, electromagnetic interference shielding, heating, transport of electrical signals and in sensors,* etc.*

The temperature of heating materials depends on the thermal power given off the textile. Clothing heated with textiles ensures an appropriate temperature gradient between the body and the environment. The required temperature gradient can be obtained either by passive or active clothing. Passive clothing is not suitable where a high amount of work is required, because layers and layers of clothing hinder the movement of the wearer. Active clothing can react with the changes in the metabolism or climate. In the past, the following were used as heating elements in active clothing: metallic heating elements, graphite elements, conductive rubbers, and a system of water heaters. The use of such heaters was subject to many limitations: increased the mass of clothing, rigidity of systems, and limited extraction of sweat, *etc*.

Textile heating elements can be manufactured from any type of textile products such as nonwoven fabric, knitted fabric, woven fabric and embroidery [23,24]. Heating elements made of nonwoven fabric have proved to be of little use owing to high electrical resistance of conductive nonwoven fabric. The resistance of heating elements made of woven fabric is lower than that of a heating element of the same dimensions made of knitted fabric due to the structure of these materials.

Heating material is the most important factor in designing heat radiating textiles. Heating elements are divided into two categories by shape: sheet type heating elements and wire type heating elements. However the voltage supplied, the temperature of heat radiated and the capacity of the battery are problems to be examined before selecting the heating material. The next consideration is whether or not the material is suitable for clothing. The basic pre-requisites for a heating element to be applied into clothing are: softness, flexibility and it being washable [25].

There are two ways to produce heated textiles: one way is to produce a technical fabric and then integrate electronic components, and the other is to produce a technical yarn with electronic features and then the textile is made of that yarn [26].

Heating elements in the shape of small patches can be used at several places in active type of clothing. These patches can be attached easily on to clothing through sewing. A heating patch element basically comprises four elements; a carrier, a heating material, bus bar to supply the power to the heating material, and a power supply. In the current research, the carrier is in the form of a knitted structure supported by an elastomeric yarn. The knitted structure provides the base platform to the heating material. A silver coated polymeric yarn of high resistance is used as a heating material and the bus bar, made of very low resistance silver yarn, is used to provide the power to the heating material. The heating element can be supplied with either alternating or direct voltage. However, use of high AC voltage is not advisable as it can cause of heart problems [27]. All the heating materials currently available in the market need high wattage power supplies. Therefore there is a need for such heating elements as discussed in the present paper, which can operate at low voltages [28].

### 1.1. Electromechanical Theory

#### 1.1.1. Electro-Thermal Relationship

The conductive silver yarn used in the present research is a 27 tex multi-filament low twisted yarn consisting of silver coated Nylon 6,6 filaments having a resistance of 10 ohms per cm, procured from Swicofil. For ease of analysis, the silver yarn can be considered to be a nylon filament coated with silver, where the silver forms an equivalent annulus cross-sectional area around the nylon yarn. Considering the density of nylon (1.15 × 10^3^ kg/m^3^ [29]), density of silver (1.05 × 10^4^ kg/m^3^ [29]) and resistivity of silver (1.59 × 10^−8^ Ω·m [29]), it can be shown that the experimental resistance value of 1000 Ω/m agrees with calculated value from the standard resistance equation of a material given by

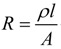
(1)

Since the electrical resistance of the metallic conductive yarn is due to the collision process [30] in the conductive area, the temperature effect on the resistance of the conductive yarn can be expressed as

*R* = *R*_0_[1+*α*(*T_t_* − *T*_0_)] (2)

#### 1.1.2. Structural Parameters

**Figure 1:**
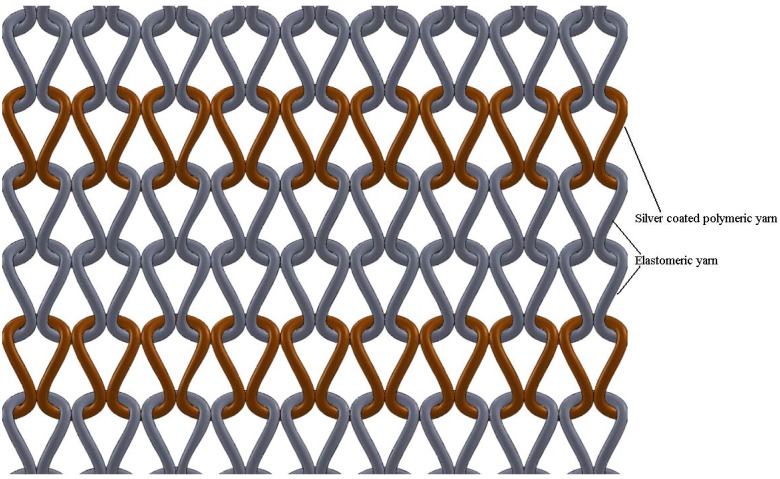
Sketch of the arrangement of the silver yarn and the elastomeric yarn in the plain knitted heating fabric.

A heating fabric that is knitted with silver coated polymeric yarn is a mesh of electrical conductors. When an electrical potential difference is applied either on two opposing sides of it or between two points, an electrical current will flow through the structure. According to electrical principles, when a potential difference is applied across a mesh, the heating effect near the terminals is observed to be less, while the current density in the middle of the fabric is higher than the edges. The effect causes the fabric to have non-uniform heating throughout its area. Due to this, in the current research, to take the maximum use of the electro-conductive silver yarn, the knitted courses of silver yarn were separated using two courses of elastomeric yarn. Due to the elastomeric yarn, the structure of the knitted silver/elastomeric mesh assumes a geometry given in Figure 1.

Due to the stretched elastomeric yarn, the silver yarn loops are pressed together at their sinker loops. The result would be that no electrical current flows in the heads of the knitted loop while the current only flows through the sinker loops.

**Figure 2:**
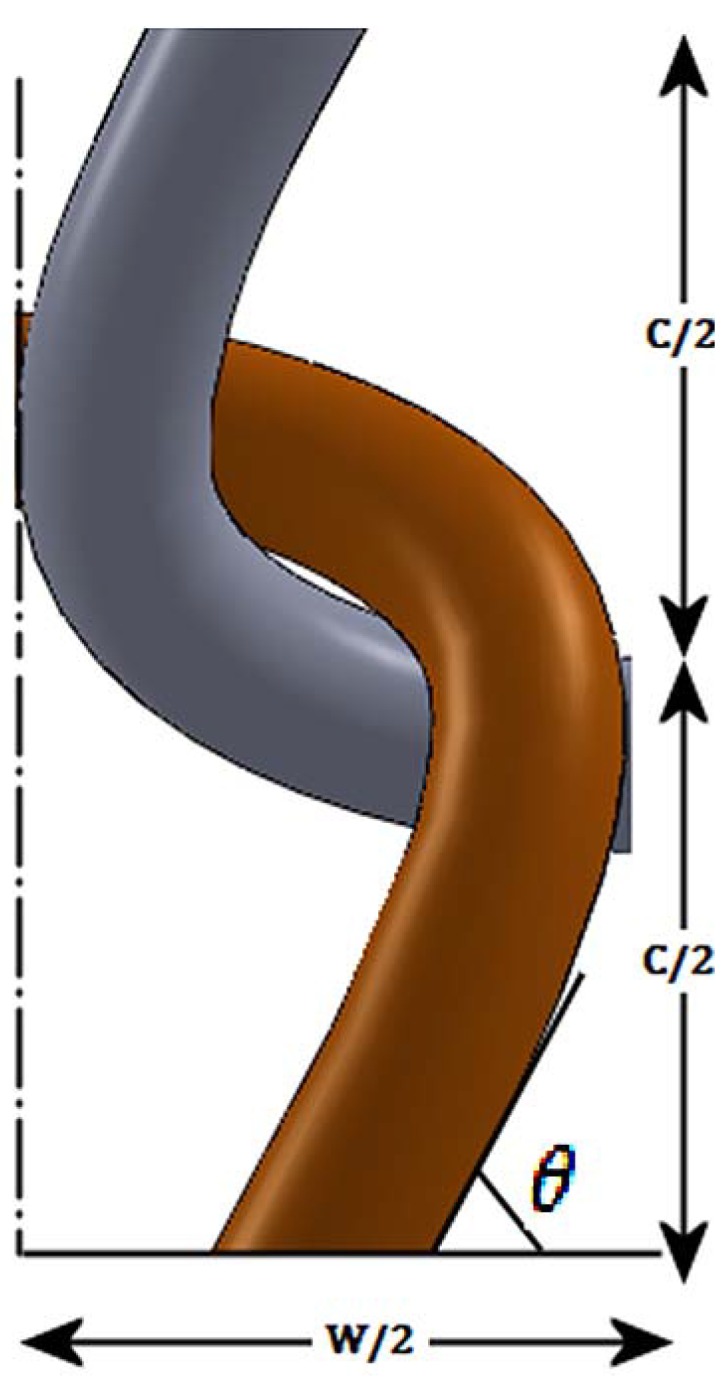
A binding point at the sinker loop of the knitted structure.

Using the simple knitted loop geometry put forward by P. Popper [31], the yarn geometry at the junction can be given by Figure 2.

According to the model developed by Popper [31], the sinker loop length for a plain knitted structure can be given by the following relationship.

For a knitted structure that is locked in the wale direction and the course direction, the electrical current flow is through the feet of the stitches in the sinker loop. From the general geometry of a stitch given in the Popper’s model [31], the approximate two dimensional yarn lengths in the sinker loop is equal to one quarter of the stitch length. Additionally, considering the yarn bend as well, the sinker loop length can be given as:

Sinker loop length per stitch

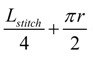
(3)
Sinker loop length for fabric width *w*

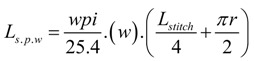
(4)

Therefore, using the above Equation (4), the fabric width required for having a specific length of sinker loop conductive silver yarn can be calculated.

Since the rib structure is a double layer structure having two planes of stitches, the sinker loop length in the rib structure can be calculated using the equation below. To reduce the complexity of the calculation, the sinker loops of the rib and interlock structures are considered to be straight yarn lengths.

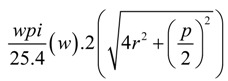
(5)

According to the same method of approximation, the interlock structure can be assumed to have two sinker loop paths. The length of a single sinker loop path can be calculated using the relationship given Equation 6. 
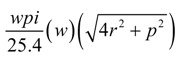
(6)

Since a single course of an interlock structure is formed by two separate yarns, the resulting equivalent resistance is half of the resistance of the single sinker loop path, assuming an equal stitch length in all three fabrics.

Assuming the power generated by the flow of electrical current through the silver yarn is completely transformed into heat energy

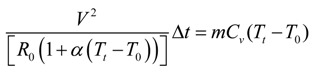
(7)

Using suitable values for the parameters in the Equation (7), for varying time “*t*” the resulting temperature T can be calculated.

In addition to above equations, the following equations are also used in simulation of MatLAB environment,

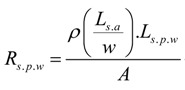
(8)

*m_silver.plain_* = *Tex_Silver_*. (Number of horizontal coarse length with in the sensing area).*L_s.p.w_* (9)

**Figure 3:**
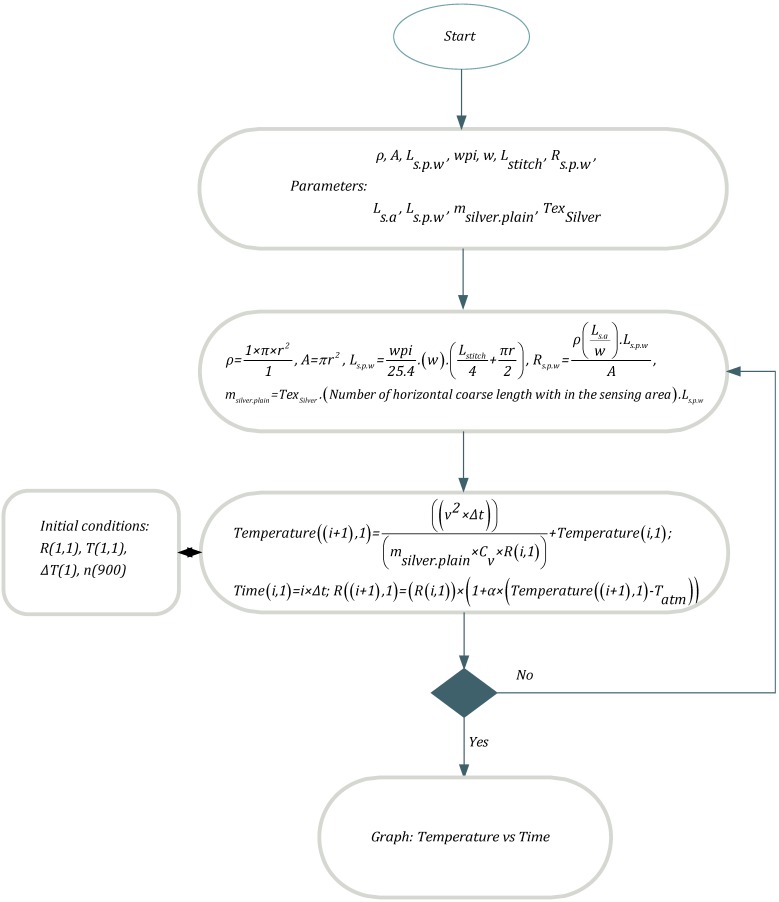
Flowchart for MatLab program.

Flow chart of Matlab program is given in Figure 3.

## 2. Results and Discussion

**Figure 4:**
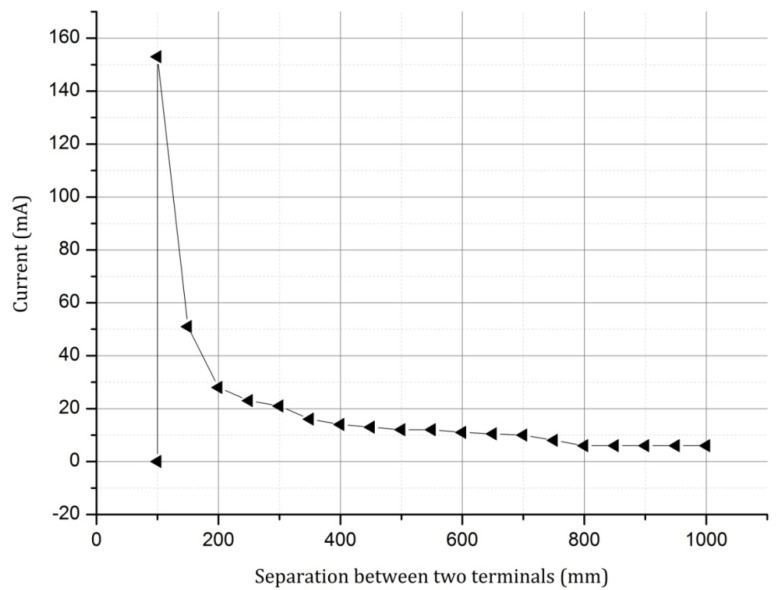
Current* vs.* conducting yarn length for the silver yarn at 9 V.

Although the silver yarn obeys the Ohm’s law, it is a polymeric yarn that has a material break down point depending on the glass transition temperature. Therefore to ascertain this point, due to the increase of current on the reduction of the conductor length (reduction of resistance), a constant voltage was applied across a silver yarn at varying terminal separation values. The resulting current* vs.* terminal separation graph is given in Figure 4.

As seen in the above graph, on reducing the conductor length, and thereby the resistance between the terminals, at the point when the current reaches the ampacity of the conductor, the conductor breaks down. Again the conductor may reach this value at a higher/lower length depending on whether there is a change in the current over time.

The conductive silver yarn and the elastomeric yarn can be generalized as a mesh of conductors. There has been previous work where a knitted mesh of conductors, either for the purpose of analyzing as a sensor or actuator, being mathematically modeled as a network of resistors using mesh analysis principles used in electrical engineering. However in the present instance, since there are two courses of elastomeric yarn between each of the knitted silver courses, the silver yarn does not represent a mesh. Instead, due to using the elastomeric yarn, the conductive yarn loops in each of the wales are short circuited at the feet of the knitted loops. Therefore, in the absence of a course direction force high enough to separate the feet of the silver yarn loops, there is no apparent change in the electrically continuous silver yarn formed by the feet (sinker loops) of the silver yarn. This feature allows a low modulus silver knitted fabric to be made with a lower effective conductive yarn path resistance than the complete yarn path. This short circuiting of the sinker loops allows a higher current to pass through the sinker loops which in turn increases the heating effect of the fabric.

**Figure 5:**
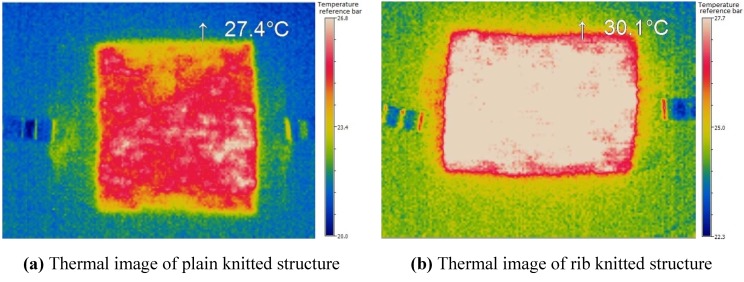

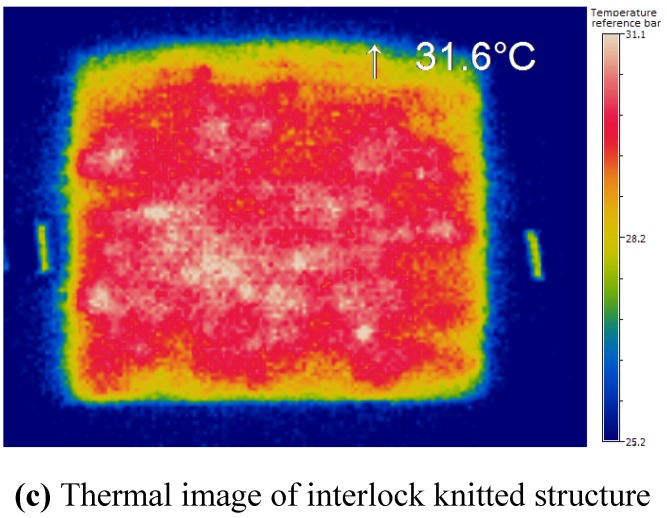
Thermal images for (**a**) plain; (**b**) rib and (**c**) interlock structures at 3 V.

In order to understand the rate of increase of temperature and heat distribution over the surface of knitted fabric, the thermal images were taken using micro-epsilon TIM160. Thermal images (Figure 5) show a uniform distribution of heat at the center of the fabric and outwardly.

**Figure 6:**
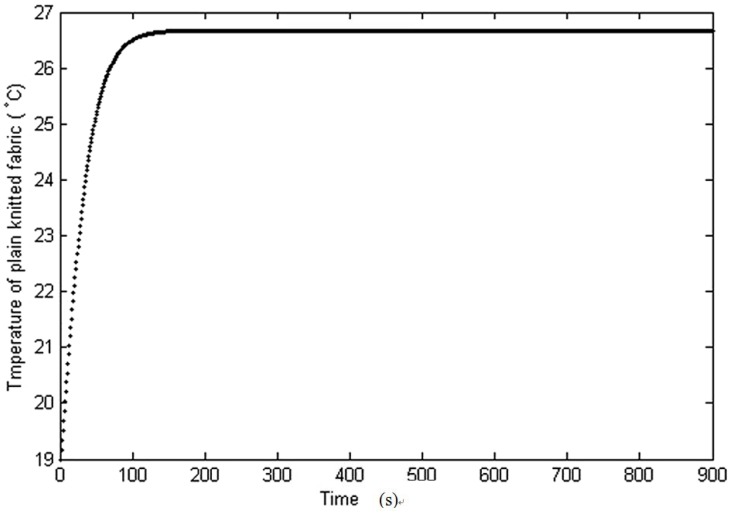
Theoretical temperature profiles of plain knitted structure.

The theoretical temperature profile (Figure 6) for the plain structure can be calculated using Equation (7) for a sinker loop length of 100 mm (fabric width 80 mm). To calculate the temperature profiles of the rib and interlock structures, for a fabric width of 80 mm, Equations (5) and (6) can be used together with Equation (7).

Since the result of using the silver yarn loops together with the elastomeric yarn loops is to present a conductive sinker loop path—to see the change in the heating/cooling effect due to the change in the sinker loop length—plain, rib and interlock structures were knitted and tested. Using Equation (4) given in the section of theoretical background, for the limiting sinker loop length of 100 mm (from Figure 4) the plain fabric width was found to be approximately 80 mm. Therefore the plain, rib and interlock structures were created to have a width of 80 mm, to observe sinker loop path lengths from 100 mm above.

**Figure 7:**
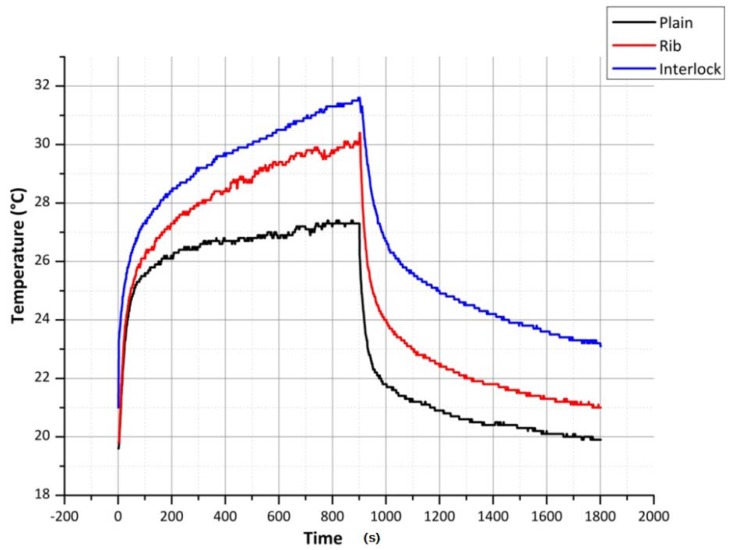
Comparison of silver yarn plain, rib and interlock structures at 3 V and 80 mm terminal separation.

As can be seen from Figure 7, compared to the sinker loop path of the plain fabric, the sinker loop path of the rib fabric is longer. This increased length, due to the increase in the resistance should produce a lower current in the rib sinker loop path. However at the same time, since the energy available in the electrical conductor due to the current flow given by *I*^2^*R*, at lower voltages, the heating effect is dominated by the yarn resistance while at higher voltages the current dominates the heating experienced of the silver yarn. This phenomenon is seen in the figures of steady state temperatures given in Table 1 plain and rib columns. However when it comes to the interlock structure, due to the lower resistance afforded by the two sinker loop paths, the current flowing through the fabric course is higher, resulting in a higher temperature. The temperature time constants for the plain, rib and interlock structures, for heating and cooling, are given in Table 2 and Table 3. As seen in Table 3, the free air cooling temperature time constants for all, plain, rib and interlock structures have close values.

**Table 1:** Steady state temperature of plain, rib and interlock structures at 80 mm terminal separation and at different voltages.

Volts	Plain	Rib	Interlock
3 V	27.4 °C	30.1 °C	31.6 °C
6 V	46.6 °C	72.2 °C	64.2 °C
9 V	83.7 °C	69.3 °C	107.8 °C

**Table 2:** Time constant of plain, rib and interlock at different length and voltages.

Volts	Plain	Rib	Interlock
3 V	297 s	501 s	642 s
6 V	567 s	568 s	534 s
9 V	390 s	99 s	307 s

**Table 3:** Time constant of cooling profile at the terminal separation of 80 mm.

Volts	Plain	Rib	Interlock
3 V	451 s	453 s	519 s
6 V	427 s	414 s	541 s
9 V	423 s	507 s	444 s

As can be seen in Figure 7, on the application of a constant voltage, the heating/cooling effect shows a transient behavior. The results of the experiments conducted to determine the speed of the heating effect for 3, 6 and 9 V is given in Table 1.

**Figure 8:**
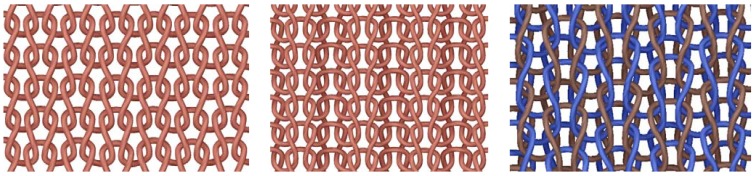
Schematic diagrams of (**a**) plain; (**b**) 1 × 1 rib and (**c**) interlock knitted structure.

The same heating fabrics were applied with successively increasing voltages and decreasing terminal separation distances to observe the polymer yarn degradation. These values are given in Table 4. The structures of these heating fabrics are shown in Figure 8.

**Table 4:** Steady state temperatures of plain, rib and interlock structures at successively increasing voltages and decreasing terminal separation distances.

Volts	Plain		Rib		Interlock	
	60 mm	40 mm	60 mm	40 mm	60 mm	40 mm
3 V	35.8 °C	39.4 °C	31.5 °C	43.7 °C	27.9 °C	31.7 °C
6 V	70 °C	86.4 °C	85.1 °C	89.5 °C	54 °C	62.9 °C
9 V	90 °C	76.2 °C	107 °C	33.8 °C	91.6 °C	49.1 °C

**Figure 9:**
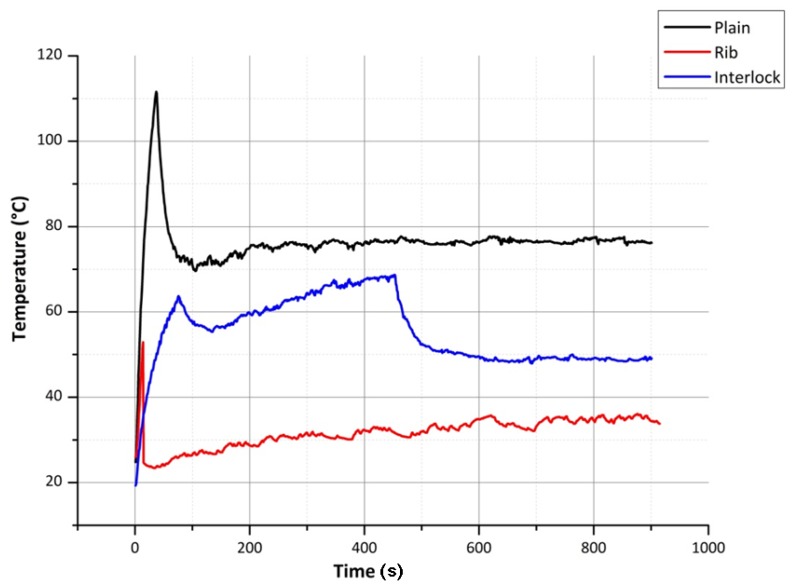
Comparison of Silver yarn, plain, rib and interlock structures at 9 V Sample size: 40 mm.

As seen in Table 4, as expected, when the voltage increases, for each type of fabric, the maximum temperature the fabric can achieve is increased. However due to the material degradation, the relationship between the plain rib and interlock structures that was seen in Table 1, cannot be seen in Table 4, especially at 9 V and at the lowest terminal separation distances (cell highlighted in third row of Table 4), the steady state temperature achieved can be seen to drop drastically (Figure 9).

Figure 7 shows one extremity of the heating performance of the knitted silver fabric where heating and cooling results from a relatively low voltage of 3 V. Figure 9 on the other hand shows the other extremity where, due to the relatively higher voltage of 9 V and very small terminal separation of 40 mm, causes a sudden increase of fabric heating and subsequent material degradation. As is apparent from Figure 9, before the degradation of the electro-conductive polymeric yarn takes place, the yarn can be seen to achieve a peak temperature. Due to the higher degradation experienced by the rib and interlock fabrics during the previous heating and cooling cycles, they were found to be highly degraded compared to the plain fabric. On observation of the samples by unraveling the knitted course paths, they were found to be broken due to overheating.

**Figure 10:**
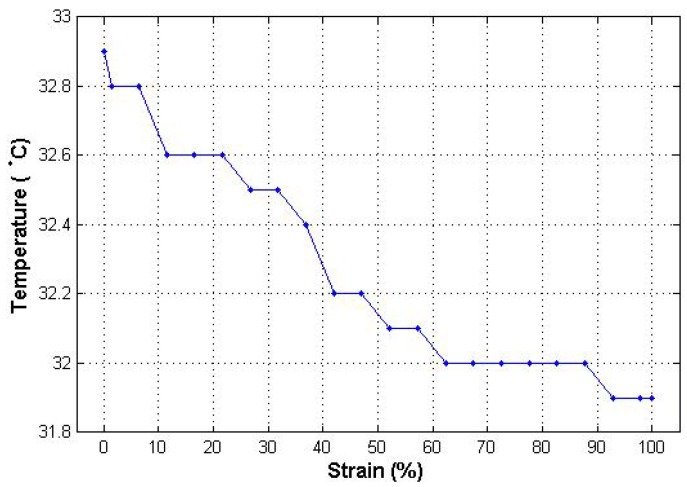
Change of temperature with respect to strain %.

According to Lam *et al.* [32], contact resistance should decrease with the increase of contact force within metal doped polymers due to the frictional abrasion. However, in this research, it was found that only a minimum amount of contact force is required for the electrical resistance to be stable. The contact resistance will not change unless there is a change in length of or cross sectional area of contacting points. As shown in Figure 10, the temperature of heating element decreases slightly with the increase in the percentage of strain. The reason for the decrease in temperature was not due to the change of electrical resistance of heating element; rather it was due to the fact that the surface area of the heating element becomes porous.

Since the focus of the present research is creating heating fabrics based on silver yarn and analyzing the data to understand the way knitted metal coated polymeric heating fabrics work, the larger part of the experiments consisted of application of controlled voltage and current to heat the heating fabric samples and the measurement of temperature using infrared heating sensors. Although every effort was made to ensure the standard laboratory conditions were maintained, slight temperature fluctuations due to the change in the laboratory heat load fluctuations are unavoidable. Due to these reasons, it was observed that the measurement of temperature was subjected to a certain degree of variation, where some of which cannot be eliminated completely. Some factors affecting the measurement of temperature are discussed below.

Since, according to Corwin and Rodenburghii [33], the amount of radiation of a heat source mainly depends on four factors. Out of these four factors, emissivity of an object is relatively difficult to control. On the use of the infrared sensors, an emissivity value needs to be input during the calibration of the sensor to account for the surface that is being measured. Although in the present case, the surface of the samples used in the experiment consisted of two different materials,* i.e.*, acrylic and silver, each of these materials consist of darker, denser and rough surface texture. Although to account for this, an emissivity value of 0.95 was used, any non-uniformity of the surface could result in the deviation of results from accurate figures.

The measurement of temperature using infrared thermometer is affected by the type of surface being examined. A flat surface does not radiate thermal energy equally in all directions. A concave surface will tend to concentrate more thermal energy into the scanned area. Similarly, a convex surface will disperse the thermal energy which will have an adverse effect on the results.

## 3. Experimental Section

The tests to measure the current and voltage parameters in relation to the heating fabrics were measured using Thurlby Thundar laboratory power supply unit (0–15 V and 4 A). The temperature sensing was carried out using a hand held infrared thermometer with dual laser targeting (Precision gold N85FR by Maplin Electronics), Calex PyroUSB temperature sensor (Pyro-USB-CF) with a distance to measurement spot size ratio of 20:1 and thermal imager TIM160 by Micro-Epsilon. The research study was conducted for knitted fabrics. The structures investigated were plain knit, rib and interlock knitted with elastomeric yarn (70 Tex double covered air mingled yarn with Lycra core and an Acrylic covering) and silver yarn was composed of a untwisted bundle of filament yarns (27 Tex silver coated polymeric yarn with 10 ohms per cm). A computerized Shima Seiki SES 122-S electronic knitting machine was used to knit the plain, rib and interlock samples for the tests conducted. The course density and wale density figures for the three types of structures are given in the table below (Table 5).

**Table 5:** Stitch density values for plain, rib and interlock heating fabrics.

Knitted structure	Courses per inch	Wales per inch
Plain	11	22
Rib	10	20
Interlock	12	12

**Figure 11:**
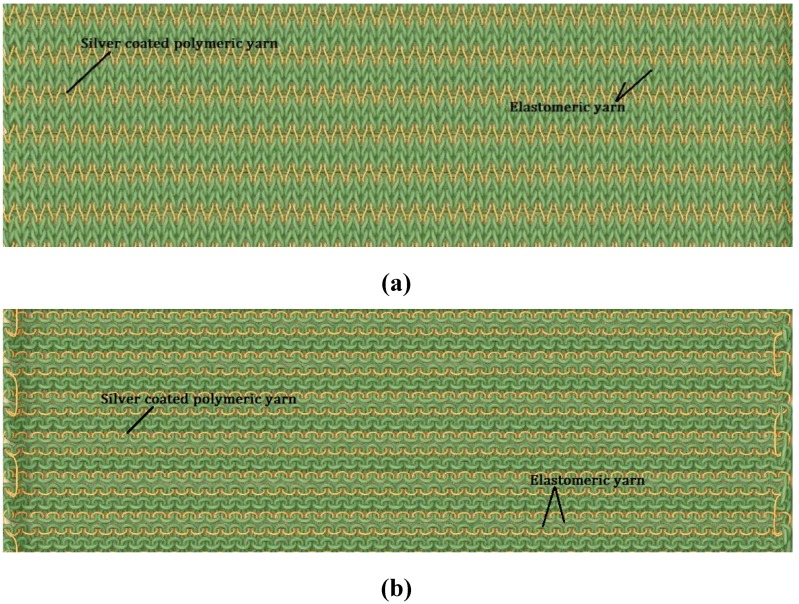
Yarn path notations for plain (**a**) technical front and (**b**) technical back.

Two courses of elastomeric yarn were knitted between each of the silver yarn courses to prevent the occurrence of electrical short circuits in the middle of the structure (Figure 11). The silver yarn stitch length values for plain, rib and interlock structures were 5.5 mm, 6 mm and 12 mm respectively. The heating fabrics were relaxed using the steam table (BM STIRO tipo type 893) to prevent any dimensional instability during the subsequent temperature measurements.

**Figure 12:**
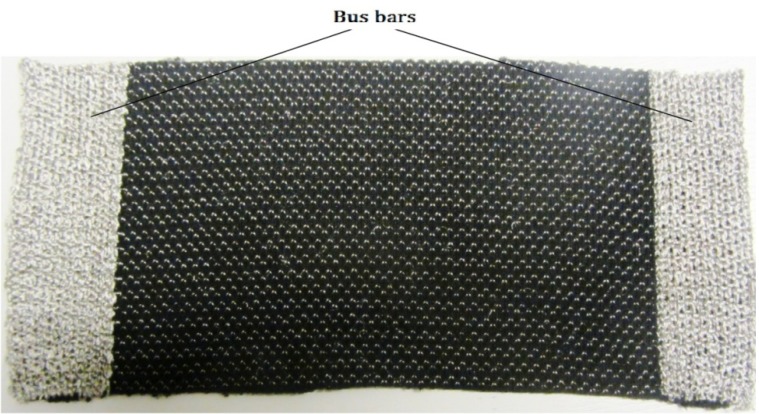
Knitted heating fabric with bus bars for electrical terminals.

The heating fabric samples were tested with both copper terminals and knitted silver bus bars on the left and right edges to supply power (Figure 12). Since on investigation, it was found that the heating fabrics with copper terminals do not have good heating distribution over all the courses, subsequently all the tests were conducted using fabrics with silver bus bars. Although the fabric structures are dense enough on visual examination, to correct for any errors introduced due to the porosity of the fabric and due to the emissivity of the surface on which the fabrics are resting, pure black surface was used under the samples during all the temperature measurements.

During the tests, the fabrics were placed on flat horizontal surfaces and the infrared temperature sensor was mounted at right angles to it at 100 mm above the fabrics to achieve a measurement spot size of 5 mm diameter, as instructed for the use of the infrared temperature sensors. Using the PyroUSB sensors, the heating temperature* vs.* time profiles were recorded from the start of the tests to the achievement of a steady state temperature level and cooling profiles up to the room temperature on the removal of power wherever relevant.

To observe the change in the heating effect due to the separation between the terminal bus bars, temperature measurements were conducted for 3 different separation values.

Generally in heating garments, where heating fabric patches are used, it is more desirable if there is no fluctuation of the heating effect, beyond the transient region of heating, due to stress and strain of the fabric. This way even if there is a reasonable amount of stretch in the fabric, the garment is able to function without any undesirable temperature fluctuations. To account for this, in the present research, the knitted structures were created in such a way that current passes through the length of the knitted silver yarns in each of the courses between the terminals. Due to this, the structure had no silver to silver knitted loop binding points between courses. Therefore to observe the presence of any variation of the heating effect of the fabrics, resulting from the stretching of the fabric within its elastic limit, experiments were conducted, according to the British standard EN ISO 2062:2009, at standard laboratory environment (65% RH and 250 °C), while recording the temperature profiles for the three silver knitted structures at the same time.

## 4. Conclusions

It was found that the fabrics knitted with silver yarn along with elastomeric yarn can generate sufficient heat to warm up the body. These fabrics can be used to manufacture personal heating garments that can generate heat in relation to applied voltage. Therefore a high input battery can be used depending upon the heating requirements. Considering Figure 6 and Figure 7, it can be seen that the steady state temperature of 26.7 °C observed for plain knitted heating fabrics, closely validates the theoretical result of 27.5 °C achieved using the theoretical model, simulated using the MATLAB program. It was observed that silver yarn degrades with the increase of voltage and with decrease of current carrying yarn length. The interlock structure knitted with silver yarn has better heating performance, to plain and rib structures. The reason for this is that the interlock structure allowed more current to pass through it at relatively lower voltages. Due to this, the interlock structure was observed to degrade before both the other structures, since the other structures do not allow as high a current as the interlock structure. For the same voltage level and fabric length, the plain structure was observed to allow the lowest electrical current to pass. This was due to the smaller stitch length of the plain structure. In all these cases, the distribution of heat over the entire fabric was observed to be uniform and devoid of hot spots. It was also observed that the stretching of knitted fabric has no significant effect on the change of heat generated by the fabric.

## List of Symbols

*α*: Temperature coefficient of resistance
*A*: Cross sectional area of silver coated polymeric yarn
*C_v_*: Heat capacity of silver of silver coated polymeric yarn
*L_s.a_*: Length of sensing area
*L_s.p.w_*: Sinker loop length for plain knitted fabric of width of *w*
*L_stitch_*: Stitch length of plain knitted structure
*m*: Mass of silver per unit yarn length
*p*: Pitch of the needle bed
*ρ*: Resistivity of silver coated polymeric yarn
*R*_0_: Initial resistance of sinker loop length of plain knitted fabric of width *w*
*R_s.p.w_*: Resistance of sinker loop of plain knitted fabric of width* w*
*Tex_silver_*: Count of silver coated polymeric yarn in Tex counting system
Δ*t*: Change in time
*T*_0_: Initial temperature
*T_t_*: Temperature at time* t*
*V*: Voltage supplied
*w*: Width of fabric
*wpi*: Wales per inch
